# Trends in Catastrophic Occupational Incidents among Electrical Contractors, 2007–2013

**DOI:** 10.3390/ijerph18105126

**Published:** 2021-05-12

**Authors:** Pouya Gholizadeh, Ikechukwu S. Onuchukwu, Behzad Esmaeili

**Affiliations:** Sid and Reva Dewberry Department of Civil, Environmental and Infrastructure Engineering, Volgenau School of Engineering, George Mason University, Fairfax, VA 22030, USA; pgholiz@masonlive.gmu.edu (P.G.); ionuchuk@masonlive.gmu.edu (I.S.O.)

**Keywords:** safety, electrical contractors, construction accidents, nature and outcome of injuries, chi-square test of independence, classification and regression trees, decision trees

## Abstract

This study used methodologies of descriptive and quantitative statistics to identify the contributing factors most affecting occupational accident outcomes among electrical contracting enterprises, given an accident occurred. Accident reports were collected from the Occupational Safety and Health Administration’s fatality and catastrophe database. To ensure the reliability of the data, the team manually codified more than 600 incidents through a comprehensive content analysis using injury-classification standards. Inclusive of both fatal and non-fatal injuries, the results showed that most accidents happened in *nonresidential buildings*, *new construction*, and *small projects* (i.e., $50,000 or less). The main source of injuries manifested in *parts and materials* (46%), followed by *tools, instruments, and equipment* (19%), and *structure and surfaces* (16%). The most frequent types of injuries were *fractures* (31%), *electrocutions* (27%), and *electrical burns* (14%); the main injured body parts were *upper extremities* (25%), *head* (23%), and *body system* (18%). Among non-fatal cases, *falls* (37%), *exposure to electricity* (36%), and *contact with objects* (19%) caused most injuries; among fatal cases, *exposure to electricity* was the leading cause of death (50%), followed by *falls* (28%) and *contact with objects* (19%). The analysis also investigated the impact of several accident factors on the degree of injuries and found significant effects from such factors such as *project type*, *source of injury*, *cause of injury*, *injured part of body*, *nature of injury*, and *event* *type*. In other words, the statistical probability of a fatal accident—given an accident occurrence—changes significantly based on the degree of these factors. The results of this study, as depicted in the proposed decision tree model, revealed that the most important factor for predicting the nature of injury (electrical or non-electrical) is: whether the source of injury is *parts and materials*; followed by whether the source of injury is *tools, instruments, and equipment*. In other words, in predicting (with a 94.31% accuracy) the nature of injury as electrical or non-electrical, whether the source of injury is *parts and materials* and whether the source of injury is *tools, instruments, and equipment* are very important. Seven decision rules were derived from the proposed decision tree model. Beyond these outcomes, the described methodology contributes to the accident-analysis body of knowledge by providing a framework for codifying data from accident reports to facilitate future analysis and modeling attempts to subsequently mitigate more injuries in other fields.

## 1. Introduction

Preventing occupational incidents in the construction industry is challenging, since this industry involves a large number of relatively small employers, multi-employer worksites, numerous hazards, and a highly mobile workforce [1,2,3]. The risk of injury for construction workers especially escalates when these workers interact frequently with electricity, as is the case for electrical contractors: An examination of the National Traumatic Occupational Fatalities (NTOF) database revealed that, although construction laborers have the highest number of fatalities among the 38 different occupations included in the database, electrical power installers and repairers have the highest rate of fatalities among all occupations [4]. 

Many of these risks manifest in the nature of the work—electrical contractors’ job entails installing and maintaining electrical systems as well as using a variety of hand tools (e.g., screwdrivers, pliers, knives, and hacksaws), power tools, and testing equipment [1]. Complicating the occupational safety of this entire population is the fact that, because of the degree of skill required, electricians often spend most of their career in one of two categories [1,5]: indoor contractors, who install conduits, connect wires, test circuits, and install and maintain lighting systems; and outdoor contractors, who work with high voltage wiring in settings ranging from the community to the consumer. Problematically, unlike other specialty trades, who have experienced a decreasing number of injuries and fatalities in recent years, the number of fatal injuries across electrical contractors has increased rapidly between 2011 to 2016 [6]. 

To better understand the nature of these occupational incidents among electrical contractors—and therefore to discover effective safety interventions to prevent injuries [7], especially fatalities—this study analyzed a large national database of occupational accidents to detect which statistically significant influential factors contribute to injuries and deaths among electrical contractors. This study has collected data from the Occupational Safety and Health Administration’s (OSHA’s) Integrated Management Information System (IMIS) accidents database from 2007 to 2013. Hence, the outcomes of this research work apply to the construction industry of the United States of America. Due to the categorical nature of accident factors (e.g., sources of injuries, event type), determining the correct values for these factors remains one of the main challenges of using historical accident data. Therefore, this study adopted a rigorous content-analysis method to ensure the reliability of final variables. The effects of influential accident factors on the fatality rates were then analyzed using the chi-square test of independence, Cramer’s V tests, and Classification and Regression Trees (CART) analysis using decision trees [8]. Given the statistical significance of the variables identified during this study, these results will help practitioners to better understand nature of accidents, design specialized training programs, consider safety during design, choose alternative means and methods of construction, identify high-risk periods of a project, help small contractors better allocate their resources, and more strategically select injury-prevention practices.

The research team conducted an in-depth literature review related to occupational incidents among electrical contractors. Since electrical contractors are extensively exposed to electrical hazards, the research team also reviewed existing studies to better understand how workers get involved in electrical accidents. Salient results of the literature review are provided here. 

## 2. Background 

### 2.1. Occupational Incidents Among Electrical Contractors 

There are limited number of studies that have investigated the nature of accidents among electrical contractors. In a 2003 study, Abudayyeh et al. [1] developed a survey based on the Bureau of Labor Statistics (BLS) safety and health statistics database to identify tasks associated with injury, illness, and fatality trends in electrical contracting. Their study revealed information on important factors contributing to (mostly non-fatal) injuries—such as sources of injuries, event types, and nature of injuries—that occurred to electrical workers between 1992 to 1998. While the results were of interest to the current study, their study faced several limitations: (1) results were based on perceptions of contractors who responded to survey and not their actual incident history; (2) geographical distribution of respondents was limited to Michigan; and (3) sample size was very small—only ten contractors responded to the survey. However, comparing the magnitude of attributing factors in their study to more recent accident data could provide perceptive discussion on accident mechanisms among electrical workers.

To investigate nature and impact of burn-related injuries on electrical utility workers, Fordyce et al. [9] reviewed 872 reports from the Electric Power Research Institute database between 1995 and 2004. The results indicated that while the numbers of burn-related accidents (including thermal, electrical, and chemical burns) were not high, such injuries resulted in a higher number of workdays lost and more serious injuries compared to other injury types. Although burn-related injuries accounted for just 3.7% of all injuries, they seemed to be costlier, representing about 13% of all medical costs.

Some studies supported by the Electric Power Research Institute (EPRI) focused on the safety and health of electric utility and power industry workers. For example, Fordyce et al. [10] analyzed neck injuries among electric utility workers from 1995 to 2007 and found higher rates of neck injuries in young males who had trade/craft worker experience. In another study, Fordyce et al. [11] investigated fatal and non-fatal injuries and injury-severity factors among electric power industry workers between 1995 and 2013 and found that fatal injuries were most commonly associated with vehicle collisions and contact with electric current. They also found risk of fatalities to be higher among line workers and that line workers experienced the second highest risk for non-fatal severe injuries, after meter readers. More recently, Volberg et al. [12] analyzed the EPRI occupational health and safety database to study injuries among electric power industry workers from 1995 to 2013. They found that while injury rates among electric power industry workers tended to decrease over the study period, rate of injuries remained high among certain high-risk workers: line workers, mechanics, young males, older welders and machinists, and female meter readers. Though each of these studies contributed to the overall body of knowledge, one limitation these studies shared was that their database only included EPRI member companies (i.e., 18 large electric power companies) and only covered a few occupations, both of which indicate that there may be selection bias in results. 

Outside of the United States, Marhavilas et al. [13] proposed a new hybrid risk assessment process for analyzing Greek public electrical power providers and identified high risk activities in this sector. Building on this initial success, Marhavilas and Koulouriotis [14] combined stochastic and quantitative risk assessment methods to build a more realistic forecasting framework for the electric power provider industry. More recently, Rosa et al. [15] evaluated the impact of personal factors and consequences of electrical occupational accidents in the primary, secondary, and tertiary sectors in Spain. They found that electrical accidents in all three sectors caused more severe consequences. Regarding personal factors, they found that workers’ sex, age, experience, nationality, and occupation significantly impacted type of accident. While contributions of these studies are significant, the data sources used in these studies are from outside of the United States, and there is a need to investigate accident reports among American electrical contractors.

### 2.2. Electrical Incidents on the Whole

Contact with electric current is a major cause of injury and death among construction workers [16,17,18,19,20,21,22]. Between 1994–2000, Census of Fatal Occupational Injuries (CFOI) data indicated that “contact with electric current” was the fourth leading cause of work-related deaths—after “falls,” “transportation incidents,” and “contact with objects and equipment” [20]. Finding innovative ways to identify, assess, and mitigate electrocution hazards in the early stages of a project would save lives and prevent injuries. 

Most studies investigating electrocution accidents have relied on reviews of accident reports. Jenkins et al. [23] investigated all fatal occupational injuries in the U.S. from 1980 to 1989. Electrocution was reported to be the fifth most frequent cause of occupational deaths in all industries in the U.S. (responsible for 7% of all deaths); however, in the construction industry, electrocution accounted for more than 15% of fatalities, making electrocution the industry’s second most frequent cause of death after falling. Furthermore, the construction industry was the only industry for which electrocution was one of the top three causes of fatalities, and about 39% of all fatal electrocutions happened in the construction industry. According to Cawley and Homce [24], electricians and their apprentices, followed by construction laborers and electrical power installers, were the most vulnerable groups to electrical fatal injuries.

McCann et al. [20] studied construction fatalities between 1992 and 1998 using the CFOI database and injury reports. Categorizing workers into electrical and non-electrical trades, they conducted several statistical analyses to find significant differences between these groups. The results revealed that working on or near “live” electric current is a major cause of injury and death among electrical accidents. To reduce the risk of these kinds of accidents, they suggested a permission process for people working on live circuits, along with use of personal locks and training sessions. The 61 non-fatal electrical injuries detailed in this study were limited to one hospital and therefore might not reveal common sources of injuries for electrical contractor’s trade. 

In another study, Janicak [21] analyzed CFOI data from 2003 to 2006 to identify influential variables involving electric current. He found that contact with overhead power lines was the most common injury event in both the construction industry (47.2%) and all other industries (43.2%). Other frequent electrocution events in the construction industry that caused fatalities included contact with wiring, transformers, or other electrical components (34.3%); and contact with the electric current of machines, tools, appliances, or light fixtures (12.4%). These were followed by some minor causes, including contact with electric current, unspecified (2.6%); struck by lightning (2.4%); and contact with underground, buried power lines (1.0%). Janicak [21] also calculated proportionate mortality ratios (PMRs) and found that the construction industry had 20% more fatalities due to contact with wiring, transformers, or other electrical components than was expected statistically. The study concluded that contact with wiring, transformers, and other electrical components contributed to a higher proportion of fatalities in the construction industry compared to other industries. Notably, Janicak’s study focused on fatal injuries and did not consider non-fatal scenarios, which are more prevalent among electrical workers.

In an attempt to develop a coding system that would facilitate the categorization of fatal electrocutions and selection of prevention strategies, Chi et al. [25] examined 255 occupational electrical deaths from 1996 to 2002. They considered variables such as the cause of electrocution, performing task, source of injury, individual factors, and company size, and identified five main accident patterns for electrocution accidents: direct worker contact with an energized power line; boomed vehicle contact with an energized power line; conductive equipment contact with an energized power line; direct worker contact with energized equipment; and improperly installed or damaged equipment. The results of this study could help practitioners determine electrocution protection strategies according to specific characteristics related to accident patterns and variables that impact potential risk factors. 

To create a safer environment near dangerous zones of power lines, Hesla [26] analyzed the underlying reasons for accidents near energized power lines and found the main contributors to be distraction of crane operators and observers, unclear working zones, and inability of workers to indicate the location of power lines. To mitigate risk of such accidents, he suggested providing appropriate equipment, such as line guards, ball markers, cone shaped markers, and line conductor coils. Other researchers developed wearable electric field sensors to notify workers or their supervisors when a worker comes in proximity to, or in contact with, a live power circuit [27]. In addition, anti-current devices that prevent transmission of electrical current from energized power lines to vehicle components [27] can be used to reduce risk of contact between a boomed vehicle and overhead power line. Alternatively, proximity and current warning devices can notify at-risk workers or operators to avoid potential contact instead of interrupting the transmission of electricity. 

As mentioned earlier, these studies focus only on electrical hazards and do not study other types of incidents (e.g., falls) in which electrical contractors may also be involved. Moreover, the data for these studies were usually collected from all trades within the construction industry (e.g., large building construction and heavy civil companies), and therefore their findings might not be pertinent to small specialty (i.e., electrical) contractors. While results of these studies can help to reduce electrocution, to be effective, safety programs need to be designed and implemented for specific trades and based on characteristics of certain tasks and sequences. Therefore, there is a need to study accident patterns among electrical contractors. 

## 3. Point of Departure 

The results of the literature review indicated four limitations in previous studies related to the occupational health and safety of electrical contractors: (1) some studies focused only on fatalities and ignored other incident outcomes; (2) most of the studies investigated electrical incidents, with only a limited number of studies examining via a large database documented incidents among electrical contractors industry-wide; (3) most of these previous studies only reported descriptive data without using any inferential statistics or machine learning algorithms; and (4) not all of accident types that can happen to electrical workers have been investigated in previous studies. 

To address these limitations, researchers need to analyze more recent incident report databases and employ more sophisticated statistical techniques to make inferences that can help practitioners better understand the nature of incidents among electrical contractors and mitigate the risk of injuries and fatalities [28]. Therefore, this study has collected data from the Occupational Safety and Health Administration’s (OSHA’s) Integrated Management Information System (IMIS) accidents database to analyze accidents related to electrical contractors using a chi-square independence test and decision trees. One advantage of using OSHA reports in this study is that the reported values for accident factors have been checked and modified based on available summaries, which serves as a major step in understanding the true mechanisms of electrical accidents. We will detail our approach in the methodology section that follows.

## 4. Methodology

To attain this study’s research objectives, the analysis first requires reliable national data of incidents among electrical contractors. Consequently, the authors acquired data from OSHA and then conducted a thorough content analysis to: (1) ensure the consistency of variables across the data, (2) reduce the ambiguity of reported values, and (3) prepare the data for statistical analysis. Previous studies have successfully used this approach to analyze construction accident databases [28,29,30,31,32]. To investigate and explain the relationship between contributing factors to accidents and the degree of accident injuries, chi-square, Cramer’s V tests, and the data mining method known as Classification and Regression Trees (CART) were applied. The rest of this section has been devoted to explaining each of these steps.

### 4.1. Incident Database 

Using the OSHA IMIS online database, the authors collected 621 accident reports about injuries involving electrical contractors between 2007 to 2013. While most of these accidents only involved one worker, some cases included multiple injuries; thus, in total, 689 electrical workers’ occupational injuries were entered into the database during the seven-year period of this study. One should note that OSHA only requires documentation of ‘catastrophic’ accidents, wherein a work-related accident caused a fatality, in-patient hospitalization, amputation, or loss of an eye. Therefore, most of the reported accidents had serious outcomes, and only a small fraction of reports included non-hospitalized injuries. Other than non-hospitalized amputations/loss of an eye (which still need reporting), two reasons for the presence of non-hospitalized cases in data include: (i) the accident affected multiple employees and therefore was reported because some injuries were fatal or needed hospitalization, and (ii) the employer reported incident even without being required by law. It is also important to note that inclusion in OSHA’s database inherently means an accident occurred. Thus, studying this database enables researchers to assess accidents that occurred historically rather assess or predict rates of accidents, which would require data outside the scope of this study.

Within each entry in the large database appears a summary of each accident, as reported by OSHA inspectors, and a limited number of variables used to describe the accident (e.g., event type, source, and cause of injury), its context (e.g., project end-use, type, cost), and its consequences (e.g., nature and degree of injuries, injured part of body). To process data, this study adopted categories found in the Occupational Injury and Illness Classification Manual (OIICM), developed by the U.S. Department of Labor Bureau of Labor Statistics [33]. In total, 64 cases were omitted from further investigation due to insufficient or missing information, leaving 619 incidents for analysis. 

### 4.2. Analysis Methodologies

#### 4.2.1. Pearson Chi-Square Test of Independence and Cramer’s V

When variables of the study are nominal, chi-square test can be used to determine significant associations among any pair of variables by calculating a test statistic (i.e., χ^2^), which approaches a chi-square distribution [34]. Researchers have used this test for more than 100 years [35]: in psychology studies that were published in six journals in 2008 alone, the results of the chi-square test were reported more than 600 times [36]. In construction research, Mustapha and Naoum [37] have utilized this test to show that the effectiveness of construction managers is related to their age, university degree, membership in professional institutes, overseas experience, and management style. Zuppa et al. [38] have performed chi-square tests on survey data and found that building information modeling have strong positive impact on projects’ success measures such as quality, cost, and schedule. Similar to these efforts, this study has adopted chi-square test of independence to identify accident factors with significant effect on the degree of an injury. Consider a contingency table with R rows, C columns and c cells, the test statistic is:

(1) $$\chi 2=\overset{c}{\underset{r=1}{\sum }}\frac{{\left(O_{r}-E_{r}\right)}^{2}}{E_{r}}$$

where *O_r_* is the number of observations in cell *r* and *E_r_* is the expected count in cell *r*:

(2) $$E_{r}=\frac{M_{R}\times M_{C}}{N}$$

where *N* is the total number of observations, *M_R_* is the row marginal for cell *r*, and *M_C_* is the column marginal for that cell. Once the test statistic is known, it can be compared to a chi-square distribution with (R − 1) × (C − 1) degrees of freedom to acquire the *p*-value. A small *p*-value can reject the null hypothesis that the variables are independent. 

To evaluate the strength of significant associations, chi-square tests usually are accompanied by the simple and widely used Cramer’s V [34,39] test introduced by Cramer [40]:

(3) $$V=\sqrt{\frac{\chi 2}{N\left[\min\left(R,C\right)-1\right]}}$$

Higher values of *V* indicate a stronger association among two variables. Using Phi coefficient (i.e., Cramer’s V with the sign of the effect), one can also measure the effects at each level of significant factors [41]. The effects of several contributing accident factors on the degree of injury would be tested through these three test statistics.

#### 4.2.2. Decision Tree Learning

A decision tree is a supervised data mining methodology widely used to uncover hidden patterns in categorical data [42,43,44,45,46,47] that can be visually represented by an inverted tree-like structure or diagram. The goal of most decision tree algorithms is to split data by minimizing the impurity of the final categories. Impurity is a general term to define how well a data set is classified in a node, and it is smallest when the node includes just one class of the response variable [48]. The splitting of the new class is intended to group the data further into more similar sub-classes and hence improves the similarities between the variables within each successive class. The successive split process continues until stop condition is reached [49]. By traversing from a root node to a leaf node and fulfilling the split conditions along the way, one can form decision rules [49] which reveal existing associations between predictor variables [50] and how they combine to predict the response variable. Since decision trees are easy to use and interpret [46,51], especially when studying the association between variables or factors [43], they have been applied in similar studies to analyze occupational accidents [31,32,44,49,50,52,53]. Common decision tree algorithms include C4.5, classification and regression tree (CART), and Chi-square automatic interaction detection (CHAID). In this study, a CART technique (decision trees) was used because most of the variables considered here are categorical [52].

##### Classification and Regression Trees (CART) Algorithm 

The CART algorithm seeks traits in predictor variables and splits the data (at the root node) into exactly two groups by means of recursive partitioning. These two classes called child nodes are formed through the algorithm’s binary process. The split condition is satisfied when the observations in a class are as homogenous as possible in terms of the response variable [54]. In the process of splitting a categorical variable (such as all the variables in the research work), there are (2^k−1^ − 1) possible splits when there are k categories. The CART algorithm chooses the best split and continues the process by splitting the two current classes each into two other new classes until a certain set threshold is reached or until no more useful splits can be achieved. At these points where the splits terminate, these nodes that cannot be split further are referred to as terminal nodes. The sum of all the observations in all the terminal nodes add up to the total number of data points in the root node and data set. At each iteration, the best split chosen by the algorithm is the split that achieves the minimum impurity of the node and is defined as:

*I(A) = Φ* [*p*(y = 1|*A*)] (4)

where *Φ* represents the measure of impurity, *A* represents any node of the tree, y is the response variable with 2 classes, *p* represents the probability of the response variable of class 1 in node *A*, and I(*A*) is impurity of node *A*. ϕ is non-negative and symmetrical and reaches its minimum value when all cases in node A are ones or zeros. When there are an equal number of ones and zeros in the node, ϕ has its maximum value. However, *Φ* can be defined in multiple ways including entropy and the Gini index. As defined below, the CART algorithm calculates the Gini index to find the impurity:

*Φ*(*p*) = 2*p*(1 − *p*) (5)

Various tree forms could be the outcome of applying various measures of impurity to classify the same data. However, with just a number of cases, the Gini index tends to yield a relatively homogeneous node with low impurity since most of the data points would have similar values for the independent variable. On the other hand, with more cases, a relatively heterogenous node would yield higher impurity since different classes of the independent variables would be somewhat evenly mixed together. This outcome is preferable for data classification instead of the outcomes resulting from algorithms that apply entropy. This is because, in the latter case, nodes are closely alike in size and homogeneity [54]. After all the nodes have been formed, the algorithm would then assign classes to the terminal nodes by calculating the proportion of classes of response variable. Hence, the node will be labelled with the class of which it has the highest proportion. For example, if the amount of 0s (in this study, non-electrical injuries) is greater than half of the total number of data points in that node, then the node will be labelled as 0 (non-electrical).

In this study, in the development of the CART, the *nature of injury* was the target (response) variable and has two categories: electrical and non-electrical. The electrical category is about 40% of the data and the remaining 60% represents the non-electrical category. On the other hand, the predictor (explanatory) variables include *the end-use, project type, project cost, source of injury, environmental factor, human factor, and cause of injury*. The data set was split into training and testing (validation) data set in the process of applying CART to the raw data set. Out of the 619 accident reports used in this analysis, a random selection of 496 (i.e., 80%) of the accident reports was used as the training data set and was trained with the CART algorithm in R [55]. The remaining 123 (i.e., 20%) was used for validation/testing. The classification and regression training (CARET) package [56] and the recursive partitioning and regression trees (RPART) package [57] in R [55] were used to develop the decision tree for: (1) predicting the nature of injuries due to an accident during an electrical project; (2) identifying the factors that are most important in predicting the nature of electrical project injuries.

##### Prediction Accuracy

In a decision tree analysis, we measure predictive accuracy by instances correctly classified. The Kappa statistic is a measure of a model’s accuracy that estimates how well the predictions of the model and the actual classifications match or agree. It estimates the extent of the model’s ability to predict better than any random classifier (e.g., predicting expected accuracy). Hence, the model’s ability to observe and predict correctly yields the Kappa statistic. It uses the observed and predicted values for each class of independent variables to estimate the model’s Kappa value which range from −1 to 1 [58]. Values less than zero indicate no agreement, 0.01–0.20 indicate none to slight agreement, 0.21–0.40 indicate fair agreement, 0.41–0.60 indicate moderate agreement, 0.61–0.81 indicate substantial agreement, and 0.81–1.0 indicate perfect agreement [59]. 

##### Precision

Precision is a measure of the proportion of the time you were right when you declared an instance (a positive). In relation to this study, the precision of the proposed decision tree model is the proportion of correct prediction of electrical injuries (true positives) to all accident reports that are predicted as electrical injuries. In other words, the precision of the model is the ratio of true positives (TP) to total number of cases predicted as positives (i.e., TP + FP). 

##### Sensitivity

Sensitivity or recall measures the proportion of actual positives that you declared were positives. With respect to this study, the sensitivity or recall of the proposed decision tree model is the proportion of correct prediction of electrical injuries (true positives) to accident reports that are actual electrical injuries. In other words, the sensitivity or recall of the model is the ratio of true positives (TP) to total number of cases that are actual positives (i.e., TP + FN). 

##### Specificity

The specificity of the proposed decision tree model is the proportion of correct prediction of non-electrical injuries (true negatives) to accident reports that are actual non-electrical injuries. In other words, the specificity of the model is the ratio of true negatives (TN) to total number of actual negative cases (i.e., TN + FP).

##### Cross Validation Analysis

Cross validation is a technic that tests how well the results of a model will generalize to new data. It involves splitting the data set into a training set and a testing set. The model is given the training set on which the training is carried out. After the training, the set-aside independent testing data set that was unseen by the model is used to evaluate the results of the training. Cross validation is carried out to reduce bias and variance in the training data set in the development of the proposed model. A k-fold cross validation involves splitting the data into k folds, and then using one fold as the testing set and the remaining k − 1 folds together as the training data set. Folds 1 through k individually gets used 1 time as the testing set and k − 1 times as part of the training set in the k different fittings of the model. In this study, the accident reports making up the training data set were split into ten folds for cross validation. This is to improve the predictive capability of the model especially on new cases [49]. The training data set contained 496 accident reports and was randomly split into ten (k) folds. One of the ten folds was in turn set aside as the sub-testing set (for validation purpose) while the remaining nine (k − 1) formed the sub-training set. In an iterative process, every one of the ten folds in turn had a chance of being the sub-testing set in only one fitting of the model and part of the sub-training set in the others. The ten-fold cross validation resulted in ten iterations/fittings/resamples, ten Kappa values, and ten (sub-testing) prediction accuracies as outlined in the results section. The average accuracy of these ten resamples was computed and used in the development of the decision tree model.

## 5. Results

The results are presented in two separate sections. First, the explanatory statistics of catastrophic accidents that have affected electrical contractors from 2007 to 2013 are presented. The emphasis is on the degree of injury as the most evident outcome of these incidents. Then, the associations between degree of injury and several variables—such as type of projects (i.e., project end-use, type, and cost), worker’s task (i.e., source and cause of injuries), and other outcomes of an accident (i.e., nature of injury, injured part of body, and event type)—are tested. 

### 5.1. Exploratory Analysis

Within the accident reports in the database, in total, 226 (37%) of accidents resulted in a fatality. The remaining (non-fatal) injuries were filed into two categories: 343 (55%) hospitalized injuries and 50 (8%) non-hospitalized injuries. As mentioned in the research methods, eight variables were coded in the content analysis to better understand the nature of accidents. The salient results and the rates of ‘degree of injury’ for each category are presented in Table 1. One should note that a *fatality rate* in this study represents a specific quantity *given the occurrence of a catastrophic accident.* Hence, this fatality rate is the share of fatal injuries from the total number of *catastrophic injuries* in the dataset and should not be confused by estimated rates that are calculated using full-time equivalent workers. In other words, a fatality rate of 37% simply means that 37% of all injury events in the data led to a death; not that 37% of electrical workers would die on the job.

As far as end-use is concerned, electrical accidents occurred mostly in building projects (77%), with commercial buildings being the dominant end-use. In non-building projects, *utility systems* (particularly power and communication lines) were the primary environments. The results indicate that different environments have quite similar fatality rates. 

Another variable that can describe the project condition is the project type. Project type, as opposed to end-use, implies the purpose of construction projects and not their context. The project types with the most accidents were *new project or new addition* (36%), *alteration or rehabilitation* (28%), and *maintenance or repair* (25%). Conditional on an accident occurring within these project types, the fatality rates for these accidents were 40%, 40%, and 35%, respectively, which are all close to the total fatality rate among electrical contractors (i.e., 37%). However, while *demolitions* represent only 2% of projects, their accidents’ fatality rate (58%) is much higher than other types of projects.

Regarding project costs, a large proportion of accidents occurred during relatively small-budget projects: around 74% of projects in have *budgets less than $500,000*. While most of cost categories present fatality-rates close to the average, two categories with the highest budgets show different values: projects with *budgets between $5 to $20 million* have significantly higher fatality-rates (i.e., 52%) while projects with *more than $20 million budgets* have fatality rates of 24%, which is much less than the average.

As far as the sources of injuries were concerned, the largest category is *parts and materials*, as it involves all the electrical parts. Regarding the degree, though, *vehicles* and *machinery* caused higher fatality rates, with 58% and 44% of accidents being fatal, respectively.

With regards to causes, installing equipment (HVAC and other), interior plumbing, ducting, electrical work, and installing plumbing, lighting fixtures were three individual causes with the most frequency. However, in terms of the severity, the fencing, installing lights, signs, etc. cases represent the highest fatality rate. 

To explain the circumstances of an accident, OSHA reports the event type. While having high proportions of *exposure to electricity* with high fatality rates was expected, the findings also show that *fall* accidents are quite prevalent among electrical contractors.

For the nature of injuries consideration, only three categories (*fractures*, *electrocutions* and *burns*) account for 72% of all injuries. However, in terms of the degree of the injuries, *electrocutions* and *concussions* have given rise to the most severe injuries.

Regarding different body parts that were injured in this data set, *upper extremities*, *head*, and *body system* were the parts with the most injuries, respectively. Injuries to the *body system* resulted in the highest fatality rate (64%), indicating how electricity can critically affect the whole body.

Other variables such as human and environmental factors were also reported by OSHA inspectors. Table 2 reports some of the more common factors (i.e., each factor represents at least 5% of the frequency after excluding the missing cases) for each variable. 

The findings show that while *misjudgment* is by far the main human factor, problems with *lockout/tagout procedures*, *inappropriate position for task*, and neglecting necessary *safety devices* were more dangerous. For environmental factors, *work surface and facility layout condition* is the most common factor among electrical workers that leads to accidents. However, *material-handling equipment or method*, *overhead moving- or falling-object action*, and *squeeze-point action* caused higher fatality rates. 

### 5.2. Chi-Square Independence Test and Cramer’s V

As mentioned earlier, the second objective of the study was to test whether the degree of injuries (i.e., fatality, hospitalized injury, and non-hospitalized injury) was associated with the type of projects, the worker’s task, or other outcomes of an accident. Table 3 shows the results of the Chi-square test for eight variables. 

Non-hospitalized and hospitalized injuries can be combined into one “Non-fatal” category—as opposed to the “Fatal” injuries—to test the effect of accident factors on degree of injury more directly. Table 4 presents the results of this test for eight variables.

The *p*-values in Table 3 and Table 4 indicate that, at the significance level of 0.05, the degree of injury is significantly affected by the type of project, sources of injury, type of accident, nature of injury, and injured part of body. Cause of injury can be considered significant only when hospitalized and non-hospitalized injuries are combined (Table 4). This suggests that for this variable the result should be interpreted more carefully. For the rest of variables, it is safe to continue with ‘fatal’ versus ‘non-fatal’ scenario as they are significant in both cases. The results of both tables, however, show a lack of evidence to claim an association between degree of injuries and end-use, nor between degree of injuries and cost of projects. The Cramer’s V values show the amount of association between the significant factors and the degree of injuries. Nature of injuries and part of body have the highest association with the degree of injury. To locate the effects among these two significant factors, the values of Phi coefficients are calculated for each level of nature and body parts (Table 5).

### 5.3. Decision Tree Analysis

#### 5.3.1. Model Interpretation

**Figure 1:**
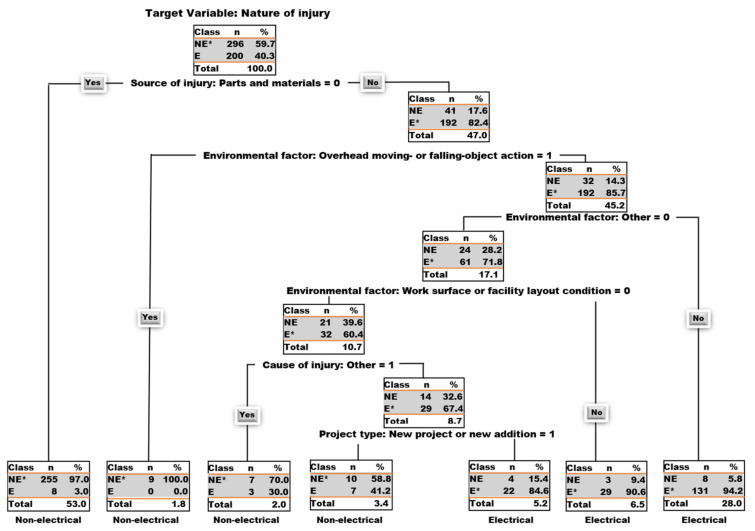
Decision tree for the prediction of nature of injury.

**Figure 2:**
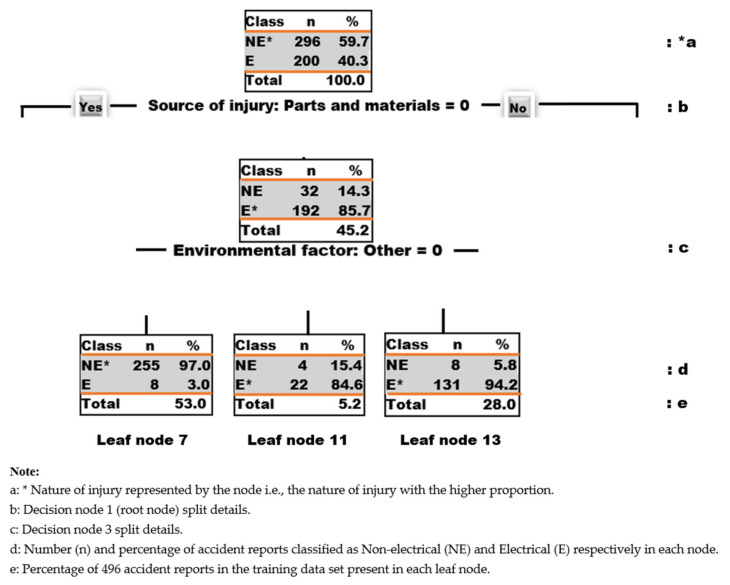
Explanatory notes on decision tree representations.

The proposed decision tree model in Figure 1 displays the classification of the nature of injury of electrical contractors as observed in the accident reports analyzed in this experiment. This tree was generated from 496 accidents reports that make up the training data set and seven project information/features/attributes namely: end-use, project type, project cost, source of injury, environmental factor, human factor, and cause of injury. The decision tree model was built for predicting the target variable which is the nature of injury. In this experiment, the nature of injury has two categories: electrical and non-electrical. The nature of injuries involving electrical burns and Electrocution (electrical shocks) are regarded as electrical and the rest (such as amputations, avulsions, enucleations, bruises, contusions, concussions, etc.) are labelled non-electrical as shown in Table 1. The tree was pruned with R tuning parameters to avoid overfitting [46] and the optimal model with the best accuracy was selected. It can be seen from Figure 1 that the decision tree model has a total of thirteen nodes of which seven nodes are leaf nodes. Figure 2 provides some explanatory notes on the decision tree model. 

By looking at Figure 1, it can be observed that out of the 496 accident reports in the training data set, a total of 463 reports (which accounts for 93.35%) were correctly classified and 33 reports (which accounts for 6.65%) were incorrectly classified. It can be seen that the target attribute in the root node is the nature of injury; the first level attribute is the source of injury as parts and material; the environmental factor labelled overhead moving- or falling-object action is the second level attribute; this is followed by the other environmental factor as the third level attribute; work surface or facility layout condition as an environmental factor is the fourth level attribute; the other cause of injury is the fifth level attribute; and lastly project type as new project or new addition is the fifth level attribute. The variable importance section below outlined the list of essential variables according to their order of importance in the development of this proposed decision tree model.

In Figure 2 below, the nodes represent split points where the observations within that node are split into the two classes: non-electrical (NE) or electrical (E). The number of observations in each node classified as NE and E are listed under the number of observations (n) for NE and E respectively. These two numbers for these two classes add up to the total number of observations in that node. In the same way, the percentage of observations in each node classified as NE and E are listed under the percentage of observations (%) for NE and E, respectively.

These two percentages for these two classes add up to 100% of the total number of observations in that node. However, the percentage total in each node represents the percentage of 496 accident reports in the training data set that is present in that node. For instance, from start, node 1 in Figure 2 has 296 NE and 200 E data points which adds up to 496 accident reports which is a 100% of the total observations or accident reports in the training data set. Hence, node 1 is labelled non-electrical (NE*) since the NE class has a higher proportion of the observations in this node. Leaf node 11 in Figure 2 has 4 NE and 22 E data points which adds up to 26 observations which is about 5.2% of the total accident reports in the training data set (i.e., 5.2% of 496 = 26). Hence, Node 11 is labelled electrical (E*) since the E class has a higher proportion of the accident reports in this node. In Figure 2, source of injury: parts and materials = 0 could be read as “if source of injury is not parts and materials”. Hence if this statement is true (Yes) you go left and if this statement is false (No) you go right. A left branch of the trees in Figure 1 and Figure 2 is always a Yes-turn while a right branch is a No-turn. In this study, nodes were counted from top to bottom, left to right, from 1 to 13. On the other hand, as seen in Figure 1, environmental factor: overhead moving- or falling-object action = 1 can be read as “if environmental factor is overhead moving- or falling-object action”. If this statement is true, you take a Yes-turn, and if this statement if false, you take a right turn. These terminologies were used in the development of the decision rules below.

#### 5.3.2. Decision Rules

Decision rules are usually formed with each leaf node of the decision tree model. Table 6 shows list of unique decision rules developed for every leaf node of the decision tree in Figure 1. The proposed decision rules are discussed further in the discussion session.

#### 5.3.3. Decision Tree Model Accuracy

The evaluation of the proposed decision tree model involved applying the model to the testing data set which contains 123 accidents reports that were set aside for validation. The results of this test are presented in the confusion matrix in Table 7. The confusion matrix compares the model predictions of the nature of injuries versus the actual classifications of the nature of injuries of the accident reports. The bold diagonal values in Table 7 show that one hundred and sixteen accident reports are correctly classified. Hence, the accuracy of the proposed decision model in predicting the nature of injury of the accidents reports in the testing data set is 94.3%. On the other hand, there are seven misclassified accident reports resulting in an error rate or misclassification rate of 5.7%.

In the confusion matrix in Table 7, one can see that true positives (i.e., accident reports involving actual electrical injuries and that are predicted to involve electrical injuries) are 45, true negatives (i.e., accident reports involving actual non-electrical injuries and that are predicted to involve non-electrical injuries) are 71, false positives (i.e., accident reports involving actual non-electrical injuries but were predicted to involve electrical injuries) are 2, and false negatives (accident reports involving actual electrical injuries but were predicted to involve non-electrical injuries) are 5. Using these values, one can calculate the precision, sensitivity (recall), and specificity resulting from applying the proposed decision tree model on the testing set as presented in Table 8. According to the evaluation results reported in Table 8, it can be seen that the proposed decision tree model in this study is dependable as it is at per with similar studies (Mistikoglu et al., 2015, Rivas et al., 2011) and as seen by an accuracy of 94.31% on new data, Kappa value of 0.881, and a *p*-Value (Acc > NIR) of 2 × 10^−16^. Hence, with 94.31% accuracy (Table 8), it successfully predicted the nature of injuries of electrical contractors into electrical injuries and non-electrical injuries using the accident reports in the testing data set.

The value obtained for the no-information rate is 0.594 and it gives an idea of the proportion of accident reports (0.596) that involve non-electrical injuries in the training data set. This means that without the proposed decision tree model and prediction is done by guessing each accident report in the testing data set to be, say, non-electrical injuries, the accuracy of prediction would be the no-information rate of 0.594 (i.e., approximately equal to the probability (0.596) of occurrence of non-electrical injuries within the training set). Also, this no-information rate of 0.594 is less than the accuracy of the proposed decision tree model presented in this study 95% of the time (i.e., 95% confidence interval of 0.886, 0.977). This means that there is a 95% chance that the true accuracy of this proposed decision tree model lies between 88.63% and 97.68%. Hence, the 94.31% accuracy of the proposed decision tree model in this study is greater and better than the non-information rate of 59.4%. The model also has a significantly better performance than chance (i.e., accuracy > no information rate) as suggested by a *p*-value less than 2 × 10^−16^. Hence it can be said that there is sufficient evidence that the accuracy of this model is greater than the no information rate with a *p*-value of <2 × 10^−16^. The kappa value of 0.88 obtained in this study indicates a perfect agreement between the classification predictions made from the proposed decision tree model and the actual classifications.

#### 5.3.4. Evaluation of Variable Importance

Unlike linear regression, all important variables may not show up on the decision tree model as a node splitter [60]. In CART, the contribution made by a predictor variable is determined by primary splits and surrogate splits. In the development of a tree, the variable that appears in the tree structure is the primary splitter, but CART also keeps track of surrogate splits and uses them as an alternative whenever the variable is missing. With the RPART package and the summary function in R, the evaluation of variable importance could be assessed as shown in Table 9. The rounded variable importance scores presented in Table 9 are scaled up to 100% as reported by the summary printout in R. Variables with an importance score less than 1 are omitted. In assigning importance to variables, the loss function (e.g., mean squared error) that can be attributed to each variable at each split is tabulated and summed. Hence, in evaluating variable importance, the goodness of split measure is summed up for each split where it is a primary variable and where it is a surrogate. Therefore, a variable that does not show up in a tree may be assigned a high variable importance depending on the measures obtained where it is a surrogate and primary splitter. Using this approach, variable masking and nonlinear correlation among variables could be revealed in the ranking of variable importance [42]. Some variables in Table 9 such as “Source of injury: Structures and surfaces;” “Human factor: Malfunction in lockout/tagout procedure;” and “Environmental factor: Overhead moving- or falling-object action” have nonzero importance in the development of the tree but still did not show up in the tree structure. This means that they played an important role in the development of the tree in Figure 1 strictly by acting as surrogates to the other splitting variables that showed up on the tree. 

By looking at Figure 1 and Table 9, one can see that *parts and materials* (root node) followed by *tools, instruments, and equipment* as sources of injury are the most relevant variables for the prediction of the nature of injury. It can also be observed that the two least important variables for predicting the nature of injury in electrical projects are *new project or new addition* project type and *other* cause of injury.

#### 5.3.5. Cross Validation Results

The cross-validation results of the training data set are presented in Table 10. The prediction accuracy of the training data set was arrived at by averaging the prediction accuracy obtained from each of the sub-testing data set (i.e., the 10 resamples in Table 10). This gives the mean training prediction accuracy of the proposed model. Summary statistics of the ten-fold cross validation is shown in Table 11. As one can see, the mean prediction accuracy of the ten-fold cross validation is 0.917, and the minimum and maximum accuracies obtained are 0.840 and 0.980 respectively. In the same way, the mean kappa value obtained in the ten-fold cross validation is 0.829, and the minimum and maximum accuracies obtained are 0.672 and 0.958 respectively. In comparison with similar studies (Mistikoglu et al., 2015, Rivas et al., 2011), these results (e.g., accuracy on new data = 94.31%, Kappa = 0.881, and *p*-Value: Acc > NIR = 2 × 10^−16^) indicate that the proposed model is reliable in predicting the nature of injuries that could occur in electrical projects.

## 6. Discussion

This study aimed to investigate which accident factors have a statistically significant effect on the outcome of accidents (i.e., degree and nature of injury) among electrical contractors. To address the objective, the authors reviewed every report manually to ensure the quality of the final data points, as the quality of data is paramount in any scientific study, without exception. This study also discusses the contributing factors to catastrophic construction accidents among electrical contractors. The results of exploratory, statistical, and machine learning analyses are discussed here. 

### 6.1. Exploratory Analysis

To reveal nature of accidents, summary statistics of 11 variables (e.g., end-use, project type, nature of injury) were reported. As noted by Lee et al. [61], safety-risk factors in projects are determined by location, type, and complexity of projects. Better understanding these influential variables will enable safety managers to strategically allocate their limited safety resources, particularly in small enterprises. As far as electrical contractors are concerned, given situations in which an accident occurred, the results show that the majority of accidents happened in *nonresidential buildings* (e.g., commercial, industrial), *new construction*, and *small projects* (i.e., $50,000 or less). Historic events do not inherently predict future events, but the statistical significance of past accident factors describe conditions in which future accidents may face added risk. Therefore, contractors who are working on these projects should plan for precautionary actions and consider larger contingencies in their budgets. 

The main source of injury is *parts and materials* (e.g., electrical parts)—representing 46% of accident sources—followed by *tools, instruments, and equipment* (19%), and *structure and surfaces* (16%). These findings are compatible with a study conducted by Abudayyeh et al. [1]: For non-fatal accidents among electrical contractors from 1992 to 1998 reported in BLS, Abudayyeh et al. found *parts and materials* as the most common source of injury (25%), followed by *structures and surfaces* (i.e., floors, walkways, or ground surfaces−19%) and all *other* sources (17%). In comparison with Abudayyeh and his colleagues’ results, this study shows a large increase in the share of *parts and materials*, which may suggest the need for more training regarding electrical sources. The share of accidents sourced in *tools*, *vehicles*, and *machinery* has also increased by 10%, 4%, and 3%, respectively, which may indicate the growing application of new tools and machines in construction and may emphasize the need for further, task-specific safety training and planning. One other notable outcome here is that due to the application of the comprehensive content analysis in this study, the other category is much smaller here (4%) compared to 17% in Abudayyeh et al. [1]. We posit that this difference can beneficially increase our understanding of accident mechanisms.

The most frequent nature of electrical contracting injuries were *fractures* (31%), *electrocutions* (27%), and *electrical burns* (14%). These are in contrast with the findings of Abudayyeh et al. [1], as that study reported “sprain and strains” (37%), “all other natures” (23%), and “cut and punctures” (13%) as the top three injury types among electrical workers. Only three cases of “strains/sprains” were reported in OSHA database, which can be attributed to the fact that while these injuries are prevalent among construction workers [62,63,64], since they usually do not lead to very serious consequences—such as permanent disability or fatality—they may have not been reported to OSHA inspectors. Indeed, this study’s findings relate to more severe injury types that might otherwise be neglected or washed out due to their relatively low frequency. Such a nuance demonstrates the benefit of focusing this study on the accidents within OSHA’s catastrophic database.

Considering body parts, the OSHA accident reports have *upper extremities* (25%), *head* (23%), and *body system* (18%) as the main injured body parts. Also, *lower extremities* and *trunk* were the two parts with the lowest frequency. Regarding severity, the chance of fatality is higher when the body system or head are injured. Further investigation showed that, most of the incidents in which the whole body was affected were cases of exposure to electricity. It’s important to note that the electricity usually enters from upper extremities (e.g., fingers, hands) and, most of the time, it’s only the magnitude of flow which differentiates between a small injury in upper extremities and a serious (usually fatal) injury in body system. In other words, injuries to upper extremities must be analyzed more carefully, especially among electrical contractors, as they can rapidly escalate to situations in which the whole body can be severely affected.

When considering only non-fatal accidents, Abudayyeh et al. [1] reported that “contact with objects” (including struck-by and caught in/between), “overexertion,” and “falls” are the most common accident types, representing 31%, 22%, and 20% of nonfatal accidents, respectively. Comparatively, in accident reports from OSHA regarding non-fatal cases, *falls* (37%), *exposure to electricity* (36%), and *contact with objects* (19%) caused most of the injuries. Putting aside “transportation incidents” (19%), the main three events for fatal accidents in BLS data were “exposure to harmful substances and environments” (50%), “falls” (21%), and “contact with objects and equipment” (7%). Among fatal cases of OSHA reports, *exposure to electricity* is also the leading event, causing exactly the same 50% of deaths followed by *falls* (28%) and *contact with objects* (19%). The order and magnitude of accident types are very similar in both studies especially in fatal cases. The share of exposure to electricity in fatal cases is much lower for the entire construction industry (18% in BLS data) which can be attributable to a much wider range of work categories in the industry compared to a more limited activities among specialty trades such as electrical contractors. This finding further emphasizes on the necessity of investigating accidents within a specific trade, since focusing one particular type of accident (e.g., exposure to electricity) can reduce the number of fatalities/severe injuries dramatically. For electrical contractors this means more electrical training on main sources and causes of exposures to electricity and ultimately decrease the more severe injuries presented in OSHA’s data. 

By using these findings from exploratory data analysis, one can start to decipher some of the more common accident scenarios among electrical workers. For instance, *electrocutions* and *burns* to *body system* and *upper extremities* often happened historically in *exposure to electricity* accidents wherein *parts and material* are the source of injury and *small*, *new*, *nonresidential buildings* are the location of accident. Though they used different methods, some studies have similarly examined the associations linking accident factors [65,66,67]. For instance, Chi et al. [66] investigated fatal fall accidents to demonstrate how different types of falls are linked to specific causes; the team then suggested several prevention measures based on strong links between a cause and its consequent accident. Chi et al. [67] also found that the source and cause of injury are significant factors in classifying accident scenarios. 

Furthermore, industry would benefit from studying the effect of different factors in fatal scenarios more. According to OSHA reports, and as we describe above, 37% of all catastrophic accidents that occurred to electrical contractors between 2007 to 2013 were fatal. Our investigation suggests when the project type is *demolition*, project budget is *between $5 million to $20 million,* sources of injury is *vehicles or machinery,* causes of injury is *fencing, installing lights and signs*, or *installing plumbing and lighting fixtures*, *or temporary work*, event type is *exposure to electricity*, injury type is *electrocutions or concussions*, and body part is *body system* or *head,* there is a higher chance that an accident lead to a fatality (i.e., at least 5% more than the average fatality rate of 37%). Also, human factors—such as *malfunctioned lockout/tagout procedures and inappropriate position for task*—and environmental factors—such as *material-handling equipment/method*, *overhead moving-/falling-object action*, *and squeeze-point action*— have contributed to fatalities at larger rates. When planning for injury prevention practices, existence of any of these factors could raise a red flag and consequently, safety managers can design customized interventions to reduce severity and frequency of incidents among electrical contractors. Other than their exploratory values in showing more hazardous situations for electrical contractors, these findings propose that the degree of injury might be affected significantly by some factors that are related to a project’s characteristic or a worker’s task. 

### 6.2. Statistical Analysis

To examine these potential associations, the research team applied chi-square independence test and found that, except for the *project end-use*, *cost*, and to a lower degree *cause of injury*, five accident factors have significant influence on the degree of an injury (Table 3 and Table 4). Ordered by their Cramer’s V values, *nature of injury* and *part of body* correlate with the degree of injury most, followed by *source of injury*, *project type*, and *event type*. Considering the effects of a single *nature of injury* on the degree of injury, this study has found that *electrocutions* and *concussions* are associated with more fatal injuries. Regarding injured *parts of body*, this study found that, among electrical workers, injuries that affect the *body system* result in fatalities at a greater rate than even injuries to *head*. Investigating accident scenarios that lead to such body-part specific injuries (e.g., electrocutions that affect the body system or head concussions) should be prioritized in future studies. Similar conclusions, with a lower level of certainty, can be made about other factors such as source of injury and event type. Thus, knowing that a factor (e.g., a specific *source of injury*) might lead to a fatality more often than another provides empirical evidence for planning decisions that would impact safety of workers on construction sites.

### 6.3. Data Mining Analysis

To illustrate the possibility of predicting the nature of injury of electrical contractors, a data mining technique (CART) was applied in this study. The algorithm was used to: classify the accident reports into categories of the response variable (nature of injury); gain insight into the relationship between some electrical project features (explanatory or predictor variables); and ascertaining their level of importance in terms of predicting the nature of injury. As earlier mentioned, the model presented in Figure 1 displays the relationship between some features of the project in the form of a decision tree. These relationships were defined in the form of decision rules and presented in Table 6. These rules/relationships could help safety managers in carrying-out risk assessment on jobsites. For instance, an example of the practical interpretation of a given rule is the decision rule derived with leaf node 7 which suggests that if the source of injury is not parts and materials, then the nature of injury is non-electrical. This could be interpreted to mean that the source of most of the electrical injuries that occur during an electrical project could be attributed to parts and materials. The proposed decision tree model emphasized the importance of parts and materials as a major source of injury by involving it in the first split condition at the root node. Additionally, according to the variable importance list in Table 9, parts and materials as a source of injury is highly important and was given a 49 importance score out of a total importance score of 100. In other words, about half of the importance score goes to parts and materials while the remaining half is shared by all other predictor variables. Hence, during electrical workers’ safety training, the best ways of handling all parts and materials associated with electrical job sites should be emphasized as this is very essential and could dramatically reduce electrical injuries on site. As seen in Table 9, structures, surfaces, tools, instruments and equipment are the other importance sources of injury that could be constantly addressed during site meetings and safety trainings. Another important variable is the environmental factor involving overhead moving- or falling-object action. Hence, it is important to highlight the need to use protective coverings to safeguard site workers from important environmental factors such as *material-handling equipment or method*, and *overhead moving- or falling-object action*. It is also very essential to protect workers from occupational injuries by making sure they adhere to all safety regulations because this would help prevent accidents from occurring. One can expect an electrical injury for about 40% of the time and a non-electrical injury for about 60% of the time in occupational hazards involving electrical projects. The remaining decision rules are all clearly stated as seen in Table 6. These rules give insight into the associations between project information (such as end-use, project type, project cost, source of injury, environmental factor, human factor, and cause of injury) that could help predict and prevent the nature of injury of electrical contractors. 

## 7. Conclusions

Electrical contractors working in the construction industry are exposed to various hazardous situations leading to high numbers of severe injuries and fatalities [68,69]. Electrical contractors have experienced a rise in occupational fatalities in recent years. Identifying statistically significant dependencies between these catastrophic outcomes and a handful of well-defined contributing factors in construction accidents offers a first step in mitigating the risks of construction accidents in this trade. Despite its importance, little has been understood regarding the contributing factors to occupational accident occurrence for small electrical contracting enterprises. To address this knowledge gap, the main objective of the present work is to study the individual effect of different contributing factors (e.g., project characteristics, sources, and causes of injury) on the degree of an injury. Our findings reveal that six factors have significant effects on fatality rates, with nature of injury and injured part of the body having the highest association and project type, source of injury, cause of injury, and event type with moderate impacts. The results of this research work are in line with previous studies and explain the association between electrical project features using accident reports from OSHA’s IMIS accidents database from 2007 to 2013 and proposed a model for predicting the nature of occupational injury of electrical contractors. The results of this study apply to the construction industry of the United States of America. The data mining technique known as CART (using decision trees) was employed in this research work to determine if the nature of injury (electrical or non-electrical) of an electrical contractor can be predicted from some project details such as end-use, project type, project cost, source of injury, environmental factor, human factor, and cause of injury. The results of this study, as depicted in the proposed decision tree model gave insight into: (1) the statistics of accident reports affecting electrical contractors; (2) predicting the nature of injuries due to an accident during an electrical project; and (3) identifying the factors that are most important in predicting the nature of electrical project injuries. 

This work discusses several contributing factors to analyze accidents that occur to electrical contractors and provides insight for current and future consideration. In particular, future studies can incorporate other important general variables, such as age and sex of the employee, time of the accident, more specific information on the specific type of accidents (e.g., height for fall accidents, and voltage for exposure-to-electricity cases). The severity of accidents also can be defined more accurately by considering more variables such as monetary cost of injuries or days away from work for non-fatal incidents. Future studies also can include more recent incidents that are available on OSHA’s database. Continuing research in this field will enable safety managers to develop personalized interventions to further reduce severity and frequency of incidents among electrical contractors.

## Figures and Tables

**Table 1:** Accident characteristics among electrical contractors.

	Variables	Frequency (% ^1^)	Degree of Injury (%)		
			Fatality ^1^	Hospitalized	Non-Hospitalized
End-use	Highway, street, and bridge	28 (5)	39	57	4
	Nonresidential building	406 (66)	36	55	9
	Other heavy and civil engineering	28 (5)	39	57	4
	Residential building	67 (11)	34	54	12
	Utility system	90 (15)	38	58	4
Project Type	Alteration or rehabilitation	173 (28)	40	52	8
	Demolition	12 (2)	58	42	0
	Maintenance or repair	155 (25)	35	57	8
	New project or new addition	222 (36)	40	50	10
	Other	57 (9)	14	83	4
Project cost	$50,000 and less	276 (45)	34	58	8
	$50,000–$250,000	115 (19)	39	56	5
	$250,000–$500,000	60 (10)	38	43	18
	$500,000–$1,000,000	45 (7)	38	56	7
	$1,000,000–$5,000,000	63 (10)	40	54	6
	$5,000,000–$20,000,000	27 (4)	52	37	11
	$20,000,000 and more	33 (5)	24	70	6
Source of injury	Machinery	45 (7)	44	56	0
	Parts and materials	286 (46)	39	52	9
	Structures and surfaces	97 (16)	31	62	7
	Tools, instruments, and equipment	118 (19)	24	66	10
	Vehicles	50 (8)	58	40	2
	Other sources	23 (4)	35	52	13
Causes	Fencing, installing lights, signs, etc.	30 (5)	53	43	3
	Installing equipment (HVAC and other)	121 (20)	40	55	6
	Installing plumbing, lighting fixtures	90 (15)	44	51	4
	Interior plumbing, ducting, electrical work	102 (17)	28	64	8
	Temporary work (building, facilities)	35 (6)	43	43	14
	Other	111 (18)	32	55	13
	Not reported	130 (21)	32	59	9
Event type	Caught in/between	38 (6)	34	58	8
	Exposure to electricity	253 (41)	44	47	8
	Fall	210 (34)	30	67	3
	Struck-by	78 (13)	37	46	17
	Other	40 (6)	23	63	15
Nature of injury	Amputations, avulsions, enucleations	21 (3)	0	71	29
	Bruises, contusions	19 (3)	16	37	47
	Concussions	44 (7)	59	41	0
	Cuts, lacerations	24 (4)	13	62	25
	Electrical burns	84 (14)	1	83	16
	Electrocutions, electric shocks	166 (27)	67	28	5
	Fractures	193 (31)	20	78	2
	Non-specified injuries and disorders	35 (6)	86	14	0
	Other	33 (5)	39	49	12
Injured part of body	Body system	110 (18)	64	32	4
	Head	141 (23)	45	48	7
	Lower extremities	60 (10)	0	93	7
	Multiple body parts	78 (13)	19	78	3
	Trunk	74 (12)	39	56	5
	Upper extremities	156 (25)	31	53	17

^1^ The percentages were rounded to the closest integer and some cases might not add up to 100%.

**Table 2:** Frequency and fatality rate for human and environmental factors.

Main Category	Subcategory	Frequency	Fatality Rate (%)
Human factors	Misjudgment in hazardous situation	207	37
	Malfunction in lockout/tagout procedure	61	53
	Safety devices removed or used inappropriately	39	39
	Insufficient or lack of personal protective equipment or clothing	35	34
	Inappropriate equipment for operation	34	35
	Inappropriate position for task	26	42
	Malfunction in securing or warning operation	26	31
Environmental factors	Work surface or facility layout condition	125	30
	Material-handling equipment or method	39	51
	Overhead moving- or falling-object action	39	49
	Temperature tolerance	18	11
	Squeeze-point action	17	47
	Flying-object action	16	25

**Table 3:** Associations between degree of injury (i.e., fatality, hospitalized injury, and non-hospitalized injury) and eight accident factors.

Variables Against Degree of Injury	Chi-Square Statistic	Degree of Freedom (D.F.)	*p*-Value	Cramer’s V
Project end-use	4.86	8	0.77	-
Project type	23.48	8	0.00	0.14
Project cost	18.78	12	0.09	-
Sources of injury	27.69	10	0.00	0.15
Causes of injury	17.42	10	0.07	-
Event type	33.51	8	0.00	0.17
Nature of injury	273.41	16	0.00	0.47
Injured part of body	111.96	10	0.00	0.30

**Table 4:** Associations between degree of injury (i.e., fatality and non-fatal injury) and eight accident factors.

Variables Against Degree of Injury	Chi-Square Statistic	Degree of Freedom (D.F.)	*p*-Value	Cramer’s V
Project end-use	0.40	4	0.98	-
Project type	16.86	4	0.00	0.17
Project cost	6.33	6	0.39	-
Sources of injury	21.49	5	0.00	0.19
Causes of injury	11.89	5	0.04	0.14
Event type	13.90	4	0.01	0.15
Nature of injury	201.18	8	0.00	0.57
Injured part of body	87.82	5	0.00	0.38

**Table 5:** The effect of different natures of injury and parts of body on degree of injury.

Variable	Level	Phi Coefficient
Nature of injury	Amputations, avulsions, enucleations	−0.14
	Bruises, contusions	−0.08
	Concussions	−0.13
	Cuts, lacerations	−0.10
	Electrical burns	−0.29
	Electrocutions, electric shocks	−0.38
	Fractures	−0.23
Part of body	Body system	−0.27
	Head	−0.10
	Lower extremities	−0.25
	Multiple body parts	−0.14
	Trunk	−0.02
	Upper extremities	−0.07

**Table 6:** Decision rules derived from the proposed decision tree model.

S/N	Node	Decision Rules
1	7	If the source of injury is not parts and materials, then the nature of the injury is non-electrical
2	8	If the source of injury is parts and materials, and the environmental factor leading to accident is overhead moving- or falling-object action, then the nature of the injury is non-electrical
3	9	If the source of injury is parts and materials, the environmental factor leading to accident is neither overhead moving- or falling-object action, nor unknown, nor work surface or facility layout condition, and the cause of injury is unknown, then the nature of the injury is non-electrical
4	10	If the source of injury is parts and materials, the environmental factor leading to accident is neither overhead moving- or falling-object action, nor unknown, nor work surface or facility layout condition, the cause of injury is not unknown, and the project type is new project or new addition, then the nature of the injury is non-electrical
5	11	If the source of injury is parts and materials, the environmental factor leading to accident is neither overhead moving- or falling-object action, nor unknown, nor work surface or facility layout condition, the cause of injury is not unknown, and the project type is not new project nor new addition, then the nature of the injury is electrical
6	12	If the source of injury is parts and materials, the environmental factor leading to accident is neither overhead moving- or falling-object action, nor unknown, but work surface or facility layout condition, then the nature of the injury is electrical
7	13	If the source of injury is parts and materials, the environmental factor leading to accident is not overhead moving- or falling-object action, but unknown, then the nature of the injury is electrical

**Table 7:** Confusion Matrix of the Testing Data Set.

	Actual/Reference	
**Prediction**	Electrical	Non-electrical
Electrical	45	2
Non- electrical	5	71

**Table 8:** Evaluation of the confusion matrix and decision tree accuracy.

Evaluation Statistics	Results
Precision	0.957
Sensitivity/Recall	0.900
Specificity	0.973
Accuracy	0.9431
95% Confidence Interval	(0.886, 0.977)
No Information Rate	0.594
*p*-Value [Acc > NIR]	<2 × 10^−16^
Kappa	0.881

**Table 9:** Variable Importance of the Proposed Decision Tree Model.

Variables/Attributes	Importance Score
Source of injury: Parts and materials	49
Source of injury: Tools, instruments, and equipment	13
Source of injury: Structures and surfaces	11
Human factor: Malfunction in lockout/tagout procedure	9
Environmental factor: Other	6
Environmental factor: Overhead moving- or falling-object action	4
Cause of injury: Interior plumbing, ducting, and electrical work	4
Environmental factor: Work surface or facility layout condition	2
Project type: New project or new addition	1
Cause of injury: Other	1

**Table 10:** Prediction Accuracy of the Ten-Fold Cross Validation.

S/N	Accuracy	Kappa	Resample
1	0.918	0.834	Fold 1
2	0.980	0.958	Fold 2
3	0.959	0.914	Fold 3
4	0.918	0.828	Fold 4
5	0.898	0.787	Fold 5
6	0.900	0.790	Fold 6
7	0.900	0.800	Fold 7
8	0.940	0.874	Fold 8
9	0.920	0.836	Fold 9
10	0.840	0.672	Fold 10

**Table 11:** Summary Statistics of the Ten-Fold Cross Validation.

S/N	Statistic	Accuracy	Kappa
1	Minimum	0.840	0.672
2	First quartile	0.900	0.792
3	Median	0.918	0.831
4	Mean	0.917	0.829
5	Standard deviation	0.038	0.078
6	Third quartile	0.935	0.865
7	Maximum	0.980	0.958

## Data Availability

All data, models, or code generated or used during the study are available from the corresponding author by request.
